# Citromycin Isolated from the Antarctic Marine-Derived Fungi, *Sporothrix* sp., Inhibits Ovarian Cancer Cell Invasion via Suppression of ERK Signaling

**DOI:** 10.3390/md20050275

**Published:** 2022-04-20

**Authors:** He Yun Choi, Ji-Hye Ahn, Haeun Kwon, Joung Han Yim, Dongho Lee, Jung-Hye Choi

**Affiliations:** 1 Department of Biomedical and Pharmaceutical Sciences, Kyung Hee University, Seoul 02447, Korea; choiheyun@khu.ac.kr; 2 Department of Oriental Pharmacy, Woosuk University, Jeonju 55338, Korea; jihyeahn20@woosuk.ac.kr; 3 Department of Plant Biotechnology, College of Life Sciences and Biotechnology, Korea University, Seoul 02841, Korea; haeun9906@hanmail.net (H.K.); dongholee@korea.ac.kr (D.L.); 4 Korea Polar Research Institute, Korea Ocean Research and Development Institute, Incheon 21990, Korea; jhyim@kopri.re.kr; 5 College of Pharmacy, Kyung Hee University, Seoul 02447, Korea

**Keywords:** citromycin, *Sporothrix* sp., ovarian cancer, invasion, MMP2 and MMP9, ERK1/2 pathway

## Abstract

Recently, microorganisms and their metabolites in the Antarctic marine environment have attracted attention as useful sources for novel therapeutics, including anticancer drugs. Here, we investigated the effects of citromycin, isolated from the Antarctic marine-derived fungus, *Sporothrix* sp., on human ovarian cancer cells. Citromycin inhibited the migration and invasion of human ovarian cancer SKOV3 and A2780 cells, but had no cytotoxic activity against them. Additionally, it inhibited the expression of epithelial–mesenchymal transition (EMT) markers and the activation of matrix metalloproteinase (MMP)-2 and MMP9. Moreover, extracellular signal-regulated kinase (ERK)-1/2 signaling was inhibited after citromycin treatment, and the ectopic expression of ERK negated the anti-invasive activity of citromycin. Our findings suggest that citromycin inhibits the migration and invasion of human ovarian cancer cells by downregulating the expression levels of EMT markers and MMP-2/9 via inhibition of the ERK1/2 pathway.

## 1. Introduction

Ovarian cancer is one of the most common gynecological malignancies and the leading cause of cancer-associated mortality in women worldwide [1]. As detection of ovarian cancer in its early stages is difficult, and 70–80% of patients are diagnosed at an advanced and highly invasive stage of the disease [2,3]. The standard therapy for ovarian cancer is surgery combined with chemotherapy, including cytotoxic platinum- and taxane-based drugs. Some targeted agents, such as the anti-vascular endothelial growth factor (VEGF) antibodies and poly (ADP-ribose) polymerase inhibitors PARPi, improve the outcomes in patients with ovarian cancer [4,5]. However, effective treatment options remain limited for highly metastatic and recurrent ovarian cancer.

Matrix metalloproteinases (MMPs) can degrade various components of the extracellular matrix (ECM) [6]. In particular, MMP2 and MMP9 can degrade collagen IV, the major ECM component, and play important roles in ovarian cancer [7,8]. MMP2 expression is increased with advancing clinical stage and metastasis [9]. Li et al. showed that increased MMP9 expression is closely related to lymph node metastasis and poor prognosis in ovarian cancer [10]. Epithelial–mesenchymal transition (EMT) allows epithelial tumor cells to lose epithelial cell–cell adhesion and polarity, resulting in the invasive properties of mesenchymal cells [11]. During EMT, many molecular changes occur: the expression levels of E-cadherin (encoded by human cadherin 1 (*CDH1*)), an epithelial cell marker, are decreased, whereas those of vimentin (encoded by human *VIM*), alpha-smooth-muscle actin (α-SMA, encoded by human actin alpha 2 (*ACTA2*)), and mesenchymal cell markers, are increased [12,13]. Likewise, low expression levels of *CDH1* and high expression levels of *VIM* and *ACTA2* in cancer cells indicate the high metastatic potential of cancers and correlate with the poor prognosis of affected patients [14,15,16]. Similar findings have been reported for ovarian cancer [17,18,19].

Compounds isolated from marine organisms have attracted attention as potential novel therapeutics. In particular, the Antarctic marine environment has rich bio- and chemo-diversity adapted to extreme cold-water habitats [20,21]. Moreover, the compounds obtained from fungal strains growing under extreme conditions, such as hypersaline, high-pressure, hypoxic, hypothermic, and dark areas, exert various pharmacological and biological activities [22]. *Sporothrix* sp. SF-7266 is a marine-derived fungus found in the Ross Sea around Antarctica. Previously, we isolated four compounds (penstyrylpyrone, sulochrin, citromycetin, and citromycin) from the *Sporothrix* sp. SF-7266 [23]. These compounds possess anti-inflammatory, antimicrobial, and antibiotic properties [24,25,26]. However, there are few reports on the anticancer effects of these compounds. Therefore, we examined the anticancer effects of these four compounds against ovarian cancer cells in this study.

## 2. Results

### 2.1. Citromycin Inhibits the Migration and Iinvasion of Human Ovarian Cancer Cells

**Figure 1:**
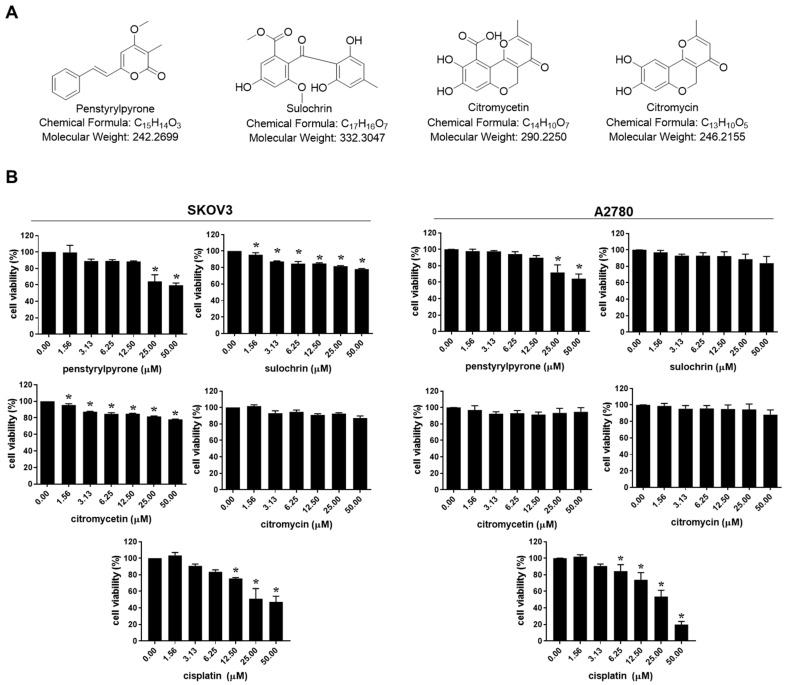
Effects of four compounds isolated from *Sporothrix* sp. SF-7266 on the viability of human ovarian cancer cells. (**A**) Chemical structure of penstyrylpyrone, sulochrin, citromycetin, and citromycin isolated from *Sporothrix* sp. SF-7266. (**B**) SKOV3 and A2780 cells were treated with the indicated concentrations of four compounds and cisplatin for 48 h and the cell viability was determined using MTT (3-(4,5-dimethylthiazol-2-yl)-2,5-diphenyltetrazolium bromide) assay. One-way analysis of variance (ANOVA) was used to identify statistically significant differences. * *p* < 0.05 versus the control group.

**Figure 2:**
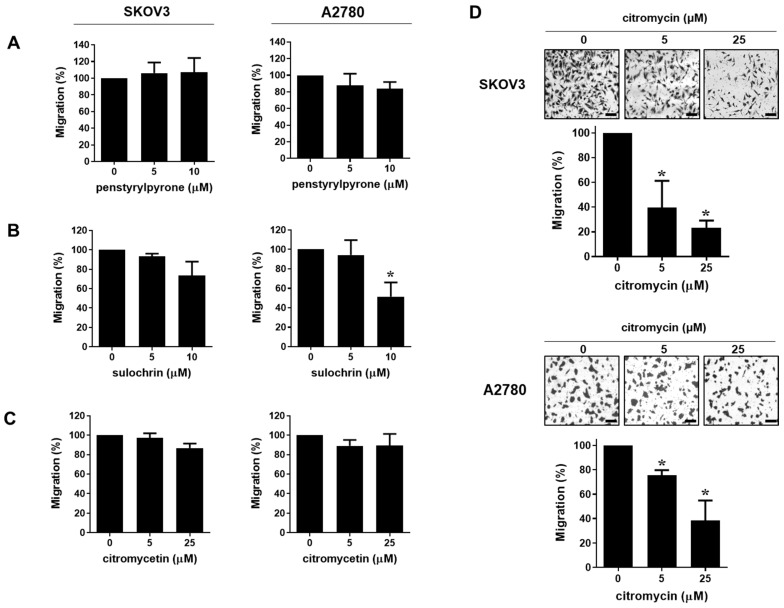
Effects of citromycin on the migration capacities of human ovarian cancer cells. SKOV3 and A2780 cells were seeded in uncoated chambers for migration assay and incubated for 24h in the presence or absence of penstyrylpyrone (**A**), sulochrin (**B**), citromycetin (**C**), and citromycin (**D**). Representative images of three independent experiments show the migratory cells. Migrated cells were stained, photographed, and counted under an inverted microscope (×100 magnification). Scale bar, 100 µm. The values represent the mean ± SD of results from three replicates. One-way ANOVA was used to identify statistically significant differences. * *p* < 0.05 versus the control group.

**Figure 3:**
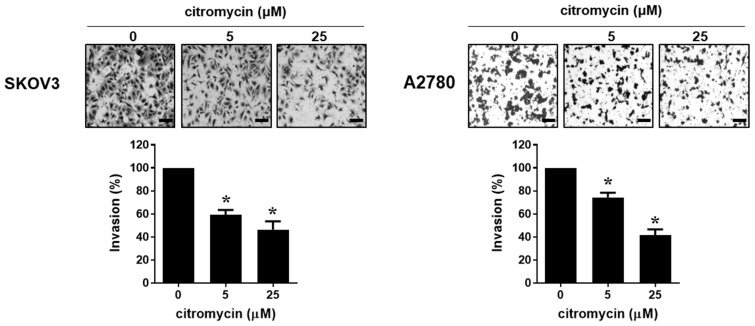
Effect of citromycin on the invasive capacities of human ovarian cancer cells. SKOV3 and A2780 cells were added in Matrigel-coated chambers for invasion assay and incubated for 48 h in the presence or absence of citromycin (5 and 25 µM). Invaded cells were stained, photographed, and counted under an inverted microscope (×100 magnification). Representative images of three independent experiments show the invading cells. Scale bar, 100 µm. The values represent the mean ± SD of results from three replicates. One-way ANOVA was used to identify statistically significant differences. * *p* < 0.05 versus the control group.

We evaluated the effects of penstyrylpyrone, sulochrin, citromycetin, and citromycin (Figure 1A), isolated from the Antarctic marine-derived fungus, Sporothrix sp. SF-7266, on the viabilities of human ovarian cancer cells. As shown in Figure 1B, the compounds only showed mild cytotoxicity against both SKOV3 and A2780 ovarian cancer cells when compared to cisplatin (half maximal inhibitory concentration (IC_50_) = 37.13 ± 26.2 µM in SKOV3 cells; IC_50_ = 27.10 ± 4.24 µM in A2780 cells). To investigate whether these compounds affected the metastatic abilities of human ovarian cancer cells, migration and invasion assays were performed after treating the cells with subcidal or non-cytotoxic concentrations of each compound. Among these compounds, only citromycin significantly inhibited the migration of both SKOV3 and A2780 cells (Figure 2). Therefore, we chose citromycin and determined its inhibitory effects on the invasive capacities of SKOV3 and A2780 cells. As shown in Figure 3, the invasive capacities of SKOV3 and A2780 cells were significantly suppressed by citromycin treatment. These data suggest that citromycin inhibits the migration and invasion of human ovarian cancer cells.

### 2.2. Citromycin Regulates MMP Activation and EMT-Related Gene Expression Levels in Human Ovarian Cancer Cells

**Figure 4:**
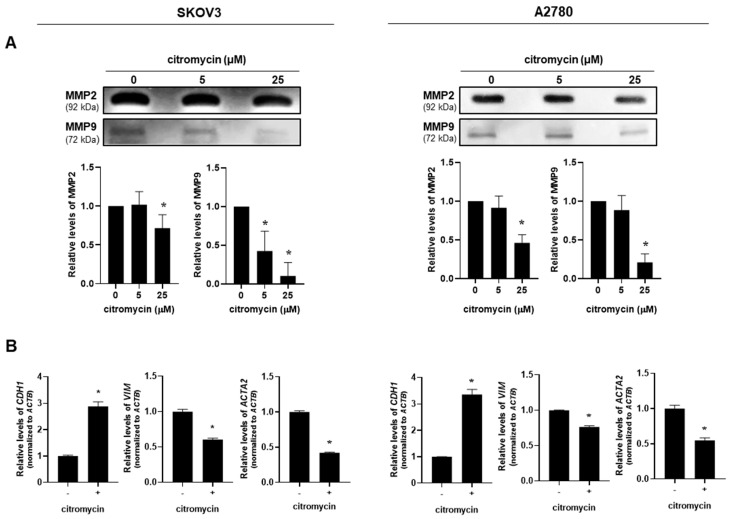
Effects of citromycin on matrix metalloproteinase (MMP) activation and expression levels of epithelial–mesenchymal transition (EMT) markers in human ovarian cancer cells. (**A**) SKOV3 and A2780 cells were treated with citromycin (5 and 25μM) for 48 h. MMP2 and MMP9 levels in the conditioned medium were examined using gelatin zymography. Densitometric evaluations of the bands are shown. Representative images from three independent experiments with similar results. The data are represented as the mean ± SD of three independent experiments. (**B**) SKOV3 and A2780 cells were treated with citromycin (25 μM) for 48 h. Real-time RT-PCR analysis was performed to measure the mRNA levels of *CDH1*, *VIM*, and *ACTA2* genes. Results were reproducible in three dependent experiments. * *p* < 0.05, as determined by one-way ANOVA (**A**) or the Student’s *t*-test (**B**).

MMP2 and MMP9 play important roles in the migration and invasion of ovarian cancer cells [7,27]. Therefore, we investigated the effects of citromycin on MMP2 and MMP9 expression levels in human ovarian cancer cells. To measure the activities of MMP2 and MMP9, gelatin zymography analysis was performed with a conditioned medium of ovarian cancer cells treated with citromycin. Citromycin (25 µM) markedly decreased the activities of MMP2 and MMP9 in SKOV3 and A2780 cells (Figure 4A). Next, we examined whether EMT was related to the inhibitory effect of citromycin on ovarian cancer cell invasion. The mRNA expression levels of the EMT marker genes, *CDH1* (E-cadherin), *VIM* (vimentin), and *ACTA2* (α-SMA), were determined in SKOV3 and A2780 cells after treatment with citromycin. Reverse transcription-polymerase chain reaction (RT-PCR) analysis showed that citromycin significantly increased the expression levels of *CDH1*, while it decreased the levels of *VIM* and *ACTA2* in SKOV3 and A2780 cells (Figure 4B). These results suggest that citromycin suppresses the migration and invasion of ovarian cancer cells by downregulating MMP2/9 activation and expression levels of EMT markers.

### 2.3. Extracellular Signal-Regulated Kinase (ERK)-1/2 Pathway Is Involved in Citromycin-Mediated Inhibition of Cell Migration and Invasion in Human Ovarian Cancer Cells

**Figure 5:**
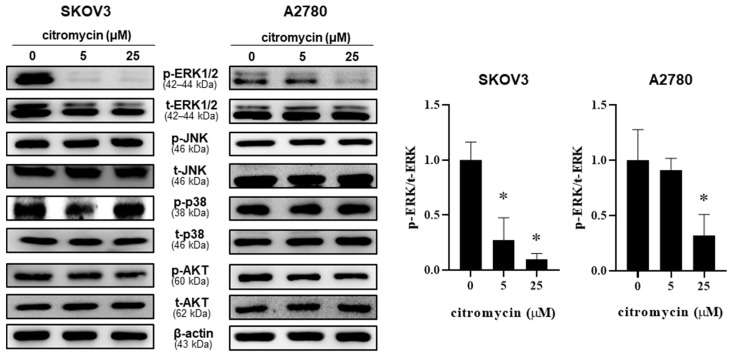
Effect of citromycin on the activation of the extracellular signal-regulated kinase (ERK)-1/2 signaling pathway in human ovarian cancer SKOV3 and A2780 cells were treated with citromycin for 48 h. The phosphorylated and total ERK1/2, JNK, p38, and AKT levels were analyzed by Western blotting. β-Actin was used as a loading control. Representative images of three independents are shown. Densitometric analysis of p-ERK and t-ERK levels are shown as the mean ± SD of three independent experiments. One-way ANOVA was used to identify statistically significant differences. * *p* < 0.05 versus the control group.

**Figure 6:**
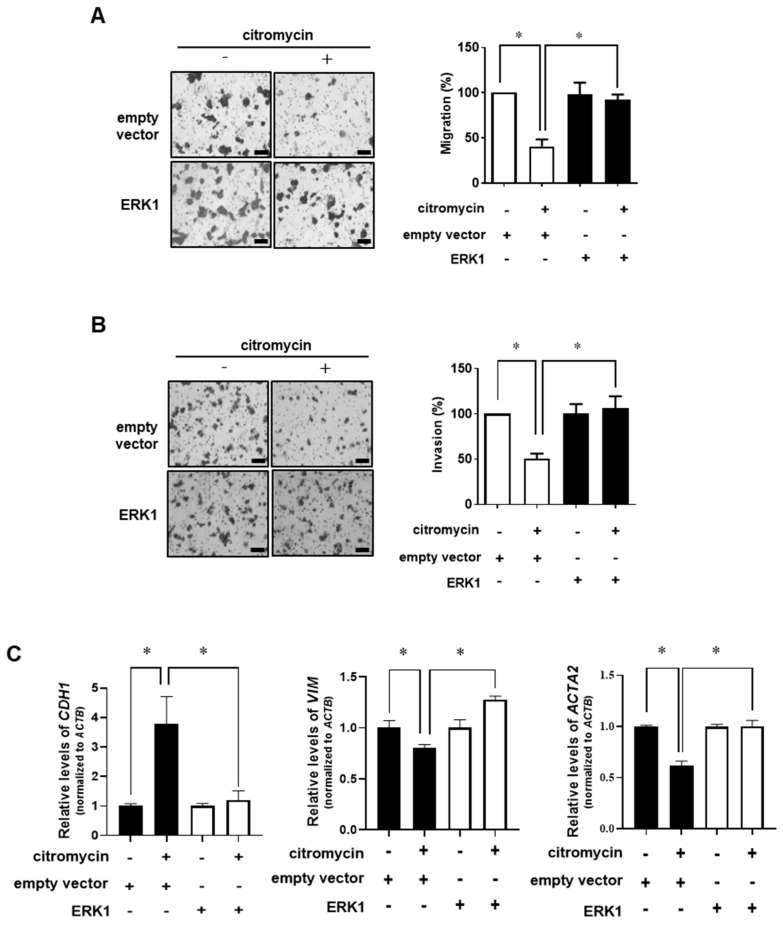
Involvement of ERK1/2 signaling in citromycin-mediated inhibition of the migration and invasion of human ovarian cancer cells. A2780 cells were transfected with ERK1 overexpressing vector or empty vector for 24 h. (**A**) The transfected cells were seeded in uncoated chambers for migration assay and incubated in the presence or absence of citromycin (25 μM) for 24 h. (**B**) For invasion assay, the transfected cells were added in Matrigel-coated chambers and incubated in the presence or absence of citromycin (25 μM) for 48 h. Representative images from three independent experiments show the migratory and invading cells. Migrated and invaded cells were stained, photographed, and counted under an inverted microscope (×100 magnification). Scale bar, 100 μm. (**C**) The transfected cells were treated with citromycin (25 μM) for 48 h. The mRNA levels of *CDH1*, *VIM*, and *ACTA2* genes were determined by real-time RT-PCR. The values represent the mean ± SD of results from three replicates. The Student’s *t*-test was used to identify statistically significant differences. * *p* < 0.05.

To explore the molecular mechanism by which citromycin modulates the migration and invasion of human ovarian cancer cells, we examined whether three members of the mitogen-activated protein kinase (MAPK) family (ERK1/2, MAPK8/JNK, and p38) and serine-threonine protein kinase B (AKT) were associated with the effects of citromycin on the migration and invasion of SKOV3 and A2780 cells. Interestingly, 25 µM citromycin significantly inhibited the activation of ERK1/2 signaling in both cell lines, while no significant effects were observed on the activation of JNK, p38, and AKT (Figure 5). These results suggest the possible involvement of the ERK1/2 pathway in citromycin-mediated inhibition of ovarian cancer cell migration and invasion. Therefore, we tested the effects of ectopic ERK1 expression on the migration and invasion of human ovarian cancer cells. We found that the citromycin-mediated inhibition of migration and invasion of A2780 cells was reversed by ERK1 overexpression (Figure 6A,B). In addition, ectopic ERK1 expression significantly rescued citromycin-induced inhibition of EMT-related gene expression. (Figure 6C). These results suggest that citromycin inhibits ovarian cancer cell migration and invasion by regulating the EMT-related gene expression levels via the ERK pathway.

## 3. Discussion

Extremophilic fungi are one of the most crucial sources of bioactive compounds, known as “extremolytes” [28]. Several extremolytes exert high anticancer activities. Deshmukh et al. extracted meleagrin from *Penicillium commune* SD-118, the fungus derived from a deep-sea sediment sample, and found that it exhibited cytotoxicity against HepG2 (human liver cancer), HeLa (human cervical cancer), Du145 (human prostate cancer), and MDA-MB-231 (human breast cancer) cell lines [29]. Tenellone H, isolated from the deep-sea fungus, *Phomopsis lithocarpus*, exhibited cytotoxicity against the human liver cancer HepG2 and human lung adenocarcinoma A549 cell lines [30].

In a previous study, we isolated penstyrylpyrone, sulochrin, citromycetin, and citromycin from the Antarctic marine-derived fungus, *Sporothrix* sp. SF-7266 [23]. Penstyrylpyrone is a styrylpyrone pigment with diverse biological activities, including anti-inflammatory activity [24]. Lee et al. isolated penstyrylpyrone from the marine-derived fungus, *Penicillium* sp. JF55, and demonstrated its anti-inflammatory effects [25]. Sulochrin has a benzophenone structure, which shows antifungal, antiviral, and antioxidant activities. Sulochrin isolated from *Aspergillus terreus* var. *aureus* shows antifungal and antibacterial activity [31]. Citromycetin and citromycin are polyketides that exhibit various biological activities, such as antibiotic (e.g., erythromycin A), antifungal (e.g., amphotericin B), anticancer (e.g., doxorubicin), immune-suppressing (e.g., rapamycin), and anti-inflammatory (e.g., flavonoids) activities [32]. Capon et al. reported the isolation of the polyketides citromycetin and citromycin from an Australian marine-derived isolate of *Penicillium bilaii* [33]. Citromycetin isolated from other *Penicillium* spp., including *Penicillium bissettii*, *P. glabrum*, and *P. setosum*, also possesses anticancer and antimicrobial activities [34,35,36]. However, the bioactivity of citromycin and its molecular mechanisms of action are largely unknown. Citromycin was first isolated from two strains of *Streptomyces* (IN-1483 and IN-2035) and its antimicrobial activity was demonstrated [37]. Citromycin isolated from the marine sponge-associated fungus, *P. erubescens*, along with other constituents, does not show any antibacterial activity against Gram-negative and Gram-positive reference or multidrug-resistant strains [38]. Thus, the bioactivity of citromycin and its molecular mechanisms of action are largely unknown. Here, we found that citromycin has anticancer activities similar to those of other well-known polyketides, such as doxorubicin [39,40]. Interestingly, citromycin inhibited the migration and invasion of ovarian cancer cells by regulating the EMT-related gene expression levels and ERK signaling activation.

*Sporothrix* sp. (Ascomycota: Ophiostomatales) has been reported to produce novel bioactive metabolites, including furofurandione, sesquiterpene, heterodimeric polyketide, and naphthalene [41,42,43,44]. For example, Krohn et al. reported the isolation of sporothriolide from *Sporothrix* sp. (strain No. 700) and its antifungal, antibacterial, and herbicidal activities [41]. The molecules 4-methyl-7,11-heptadecadienal and 4-methyl-7,11-heptadecadienoic acid, first isolated from liquid cultures of *Sporothrix flocculosa* and *Sporothrix rugulosa,* show antimicrobial activity [44]. It is noteworthy that some metabolites from *Sporothrix* sp. have antitumor activity. Chlovalicin, an ovalicin derivative isolated from *Sporothrix* sp. FO-4649, dose-dependently inhibited the growth of B16 melanoma cells (IC_50_ = 38 μM) [42]. Sporothrins B was isolated from the mangrove endophytic fungus *Sporothrix* sp. (#4335) and exhibited modest antitumor activity with an IC_50_ values of approximately 50 μM [43]. All four compounds tested in this study showed relatively low cytotoxicity against human ovarian cancer cells (IC_50_ > 50 μM). However, 5 and 25 μM citromycin markedly inhibited the migration and invasion of human ovarian cancer cells. The inhibitory effect of citromycin on the migration of human ovarian cancer cells was comparable to that of galnuisertib, a potent inhibitor of TGFβ signaling, which is well known to activate cancer cell EMT, migration, and invasion (Figure S4). To the best of our knowledge, this is the first report to demonstrate the antimetastatic activity of metabolites from *Sporothrix* sp.

Metastasis is the major cause of high mortality in ovarian cancer [45]. Unlike most solid cancers, the lack of a physical barrier between the ovary and intraperitoneal cavity facilitates the metastasis of ovarian cancer [46]. Recent studies have demonstrated certain anticancer strategies to inhibit the expression of pro-invasive factors, MMP2 and MMP9, in ovarian cancer using natural compounds. For example, lancemaside A, isolated from *Codonopsis lanceolata*, inhibits the metastasis of ovarian cancer cells and decreases the MMP2 and MMP9 expression levels [47]. Moreover, 3,4′,7-O-trimethylquercetin, a quercetin derivative, inhibits the invasion of ovarian cancer cells by suppressing the expression of uPA and MMP2 [48]. Similar to most MMPs, MMP2 and MMP9 are secreted as latent precursors (proMMP2 and proMMP9, respectively), which are proteolytically activated in the extracellular space. Thus, to inhibit ovarian cancer cell invasion, it is important to regulate the activation and expression levels of MMP2 and MMP9 [49]. In this study, zymography showed that citromycin inhibited the activities of MMP2 and MMP9. Therefore, it may be used as a potential therapeutic candidate for the treatment of malignant ovarian cancer. Additionally, we found that the inhibitory effect of citromycin on ovarian cancer cell invasion was associated with ERK1/2 signaling. Our finding is consistent with previous studies showing a relationship between the invasion of various types of cancers, including ovarian cancer, and the ERK1/2 signaling pathway [50,51,52]. For example, Moulik et al. showed that ERK1/2 inhibition significantly suppressed the activities of MMP2 and MMP9, as well as the invasion of MCF-7 and MDA-MB-231 breast cancer cells [53].

Recently, natural products isolated from marine organisms, including marine-derived fungi, have attracted attention as potential novel anticancer therapeutics. A wide range of marine natural products have been reported to exert anticancer activities through induction of cytotoxicity and apoptosis [54]. However, their antimetastatic activities are largely unknown. This study demonstrates the anti-migratory and anti-invasive effect of citromycin and provides insights for the development of novel therapeutic drugs targeting cancer cell migration and invasion to aid in the treatment of highly metastatic ovarian cancer. In addition, we showed that citromycin inhibited the invasion of ovarian cancer cells by downregulating the EMT marker gene expression levels and MMP2/9 activation via the ERK signaling pathway (Figure S5). This is the first study to demonstrate the anticancer activity of citromycin and its molecular mechanism of action, suggesting its potential to be used as a therapeutic candidate for the treatment of invasive ovarian cancer.

## 4. Materials and Methods

### 4.1. Preparation of Compounds

The isolation and structural identification of the compounds (penstyrylpyrone, sulochrin, citromycetin, and citromycin) from the Antarctic marine-derived fungus *Sporothrix* sp. SF-7266 (Ross sea, S 76°06.256′, E 169°12.675′) has been reported previously [23]. Before the compounds were evaluated for the biological activities, the purity test was performed by LC/MS methods (Figures S1–S3).

### 4.2. Cell Culture

Human ovarian cancer cell lines SKOV3 and A2780 were originally obtained from the American Type Culture Collection (HTB79, Manassas, VA, USA) and European Collection of Authenticated Cell Cultures (93112519, Salisbury, UK), respectively. Cells were cultured in Roswell Park Memorial Institute (RPMI) 1640 supplemented with 5% fetal bovine serum (FBS), streptomycin sulfate (100 μg/mL), and penicillin (100 U/mL). RPMI 1640, FBS, penicillin, and streptomycin sulfate were obtained from Life Technologies Inc. (Grand Island, NY, USA).

### 4.3. MTT Assay

The cell viability was assessed using MTT (3-(4,5-dimethylthiazol-2-yl)-2,5-diphenyltetrazolium bromide, Molecular Probes Inc. Eugene, OR, USA) assay. Cells were seeded into 96-well plates at a density of 8 × 10^4^ cells/well. After 24 h, the cells were treated with the compounds (penstyrylpyrone, sulochrin, citromycetin, and citromycin) or cisplatin at various concentrations (1.56–50 µM) and incubated for 48 h. The plates were incubated for an additional 4 h after 25 μL of MTT (5 mg/mL stock solution) was added into each well. To dissolve formazan blue, DMSO was added into each well, and absorbance was measured at 540 nm.

### 4.4. Western Blotting

Total cell lysates were prepared with protein lysis buffer (Intron Biotechnology, Seoul, Korea) supplemented with 1× protease inhibitor cocktails and PMSF (Sigma-Aldrich, St. Louis, MO, USA). The lysates were suspended with sample buffer containing sodium dodecyl sulfate (SDS) and boiled for 7 min for denaturation. Proteins were separated on 8% or 12% SDS-polyacrylamide gels electrophoresis, and then transferred onto a polyvinylidene fluoride (PVDF) membranes. The membrane was incubated with primary antibodies in Tris-buffered saline containing 0.1% Tween-20 (TBS-T) for overnight at 4 °C. Primary antibodies for p-ERK1/2, t-ERK1/2, p38, p-JNK, t-JNK, AKT, and β-actin were purchased from Santa Cruz biotechnology Inc. (Santa Cruz, CA, USA). p-p38 and p-AKT antibodies were obtained from (Cell Signaling Technology Inc., Danvers, MA, USA). The membrane washed with TBS-T to remove primary antibody, followed by incubation with specific secondary antibody (Jackson ImmunoResearch Laboratories Inc., West Grove, PA, USA) for 2 h. The signals were visualized using enhanced chemiluminescence (ECL; Abclon, Seoul, Korea) and an Image Quant LAS-4000 (Fujifilm Life science, Tokyo, Japan).

### 4.5. Gelatin Zymography

Gelatinase activity of MMP2 and MMP9 was measured by zymography. Conditioned media were collected from A2780 and SKOV3 cells treated with citromycin for 48 h. First, collected medium were concentrated using Microcon Centrifugal Filter devices (Merck Millipore, Billerica, MA, USA). Concentrated proteins were mixed with 5xnon-reducing sample buffer and electrophoresed on 6% (*w*/*v*) SDS–polyacrylamide gel supplemented with 1% (*w*/*v*) gelatin on ice. Gels were then washed twice using 2.5% Triton X-100 at room temperature to remove SDS, followed by incubation with collagen buffer at 37 °C for 24 h. The gels were stained with Coomassie blue R-250 for 50 min, and destained with destaining solution.

### 4.6. Migration and Invasion Assay

For migration assay, a 24-well trans-well unit (8 μm pore size) with polyvinylpyrrolidone-free polycarbonate (PVPF) filters was used. The cells (8 × 10^4^) were resuspended with RPMI 1640 containing 1% FBS and added in the upper part of the insert. Medium containing 5% FBS was added outside the inserts. The cells were treated with compounds for 24 h at 37 °C. For invasion assay, the filters were coated with Matrigel at a concentration of 1 μg/mL. RPMI 1640 containing 5% FBS was added to the bottom chamber. Cells were resuspended with RPMI 1640 containing 1% FBS, treated with compounds, and incubated for 48 h to invade through the Matrigel barrier. The cells that had migrated or invaded to the underside of the chamber were fixed with methanol, stained with crystal violet (0.05%) for 30 min, and photographed. The migrated and invaded cells were counted in at least five randomly chosen fields under an inverted microscope.

### 4.7. Transfection

For ERK expression, GFP-ERK1 was obtained from Addgene (Cambridge, MA, USA). A2780 cells were transfected with GFP-ERK1 vector or empty vector using Lipofectamine™ 2000 transfection reagent (Invitrogen, Carlsbad, CA, USA) under the manufacturer’s recommended procedure.

### 4.8. Real-Time Reverse Transcription PCR (RT-PCR)

Cellular total RNA was prepared with Easy Blue kits (Intron Biotechnology, Seoul, Korea) under the manufacturer’s instructions. A first-strand cDNA synthesis kit (Amersham Pharmacia Biotech, Oakville, ON, Canada) was used for reverse transcription. We used a SYBR Premix ExTaq™Kit (TaKaRa, Kyoto, Japan) and gene-specific primers. All real-time PCR assays were performed on Thermal Cycler Dice Real Time PCR System (TaKaRa). The National Biotechnology Information Center (NCBI) Primer-BLAST tool was used for primer sequence search. The primer sequences used in the real-time RT-PCR were as follows: for *CDH1* sense primer, 5′-GTGCATGAAGGACAGCCTCT, and anti-sense primer, 5′-TGGAAAGCTTCTCACGGCAT; for *VIM* sense primer, 5′-GGACCAGCTAACCAACGACA, and anti-sense primer, 5′-AAGGTCAAGACGTGCCAGAG; for *ACTA2* sense primer, 5′-CCTATCCCCGGGACTAAGACG, and anti-sense primer, 5′-AGAGCCATTGTCACACACCA; for *ACTB* (β-actin) sense primer, 5′-CCTCGCCTTTGCCGATCC, and anti-sense primer, 5′-CGCGGCGATATCATCATCC. Relative quantification of mRNA expression was carried out using the comparative CT (cycle threshold) method. Mean Ct of the gene of interest was normalized with the mean Ct of a control gene, ACTB (β-actin).

### 4.9. Statistical Analysis

The data are presented as the mean ± SD. The Student’s *t*-test or one-way ANOVA were used to identify statistically significant differences. * *p*-values < 0.05 represents significant.

## Supplementary Materials

The following supporting information can be downloaded at: https://www.mdpi.com/article/10.3390/md20050275/s1. Figure S1: The LC/MS condition for the purity test; Figure S2: UPLC-PDA/UV (254 nm)/ELSD/MS chromatogram of penstyrylpyrone (A), sulochrin (B), citromycetin (C), and citromycin (D); Figure S3: MS spectrum of penstyrylpyrone (A), sulochrin (B), citromycetin (C), and citromycin (D); Figure S4: Comparison of the effects of citromycin and galnuisertib on the migration capacities of human ovarian cancer cells; Figure S5: A schematic diagram summarizing the effect of citromycin on the migration and invasion of human ovarian cancer cells.
