# Supplementary material for: Citromycin Isolated from the Antarctic Marine-Derived Fungi, Sporothrix sp., Inhibits Ovarian Cancer Cell Invasion via Suppression of ERK Signaling
Source: Mar Drugs. 2022 Apr 20;20(5):275. doi: 10.3390/md20050275 (PMC9143255; doi:10.3390/md20050275)
Supplement: Supplementary file 1 [file marinedrugs-20-00275-s001.zip › marinedrugs-1678549-supplementary.pdf]

Supporting information for

Citromycin isolated from the Antarctic marine-derived fungi, *Sporothrix* sp., inhibits ovarian cancer cell invasion via suppression of ERK signaling

He Yun Choi [1], Ji-Hye Ahn [2], Haeun Kwon [3], Joung Han Yim [4], Dongho Lee [3], and Jung-Hye Choi [1,5,*]

1 Department of Biomedical and Pharmaceutical Sciences, Kyung Hee University, Seoul 02447, Republic of Korea; choiheyun@khu.ac.kr (H. Y. C.)

2 Department of Oriental Pharmacy, Woosuk University, Jeonbuk 55338, Republic of Korea; jihyeahn20@woosuk.ac.kr (J. -H. A.)

3 Department of Plant Biotechnology, College of Life Sciences and Biotechnology, Korea University, Seoul 02841, Republic of Korea; haeun9906@hanmail.net (H. K.); dongholee@korea.ac.kr (D. L.)

4 Korea Polar Research Institute, Korea Ocean Research and Development Institute, Incheon 21990, Republic of Korea; jhyim@kopri.re.kr (J. H. Y.)

5 College of Pharmacy, Kyung Hee University, Seoul 02447, Republic of Korea

* Correspondence: jchoi@khu.ac.kr (J. -H. C.); Tel.+82-2-961-2176

Supporting Information Contents

Figure S1. The LC/MS condition for the purity test.

Figure S2. UPLC-PDA/UV (254 nm)/ELSD/MS chromatogram of penstyrylpyrone (A), sulochrin (B), citromycetin (C), and citromycin (D).

Figure S3. MS spectrum of penstyrylpyrone (A), sulochrin (B), citromycetin (C), and citromycin (D).

Figure S4. Comparison of the effects of citromycin and galnuisertib on the migration capacities of human ovarian cancer cells.

Figure S5. A schematic diagram summarizing the effect of citromycin on migration and invasion of human ovarian cancer cells

Waters UPLC acquity

- Detector : PDA, ELS detector
- Column: Waters Acquity UPLC BEH C18 (2.1 × 100 mm., 1.7 μm)
- Column temperature : 35°C

- Mobile phase A : 0.05% FA in HPLC Water
  B : HPLC Acetonitrile

- Flow rate : 0.3 mL/min
- Injection vol : Extract- 2 uL

- Citromycin, sulochrin, and penstyrylpyrone

Time (min)	Flow rate (mL/min)	% A	% B
0.00	0.3	80.0	20.0
7.00		0.0	100.0
8.50		0.0	100.0
8.60		80.0	20.0
10.00		80.0	20.0

- Citromycetin

Time (min)	Flow rate (mL/min)	% A	% B
0.00	0.3	95.0	5.0
7.00		50.0	50.0
7.10		0.0	100.0
8.50		0.0	100.0
8.60		95.0	5.0
10.00		95.0	5.0

Thermo Scientific™ LCQ Fleet™ Ion Trap Mass

Mass range	m/z 50-2000
Scan mode	Positive & Negative
Normalized collision energy	35.0
Sheath gas flow rate (arb)	50.0
Aux gas flow rate (arb)	30.0
Sweep gas flow rate (arb)	0.0
Spray voltage (kV)	3.5
Capillary Temp (C)	275.0
Capillary voltage (V)	35.0
Tube lens (V)	50.0

Figure S1. The LC/MS condition for the purity test.

(A) penstyrylpyrone

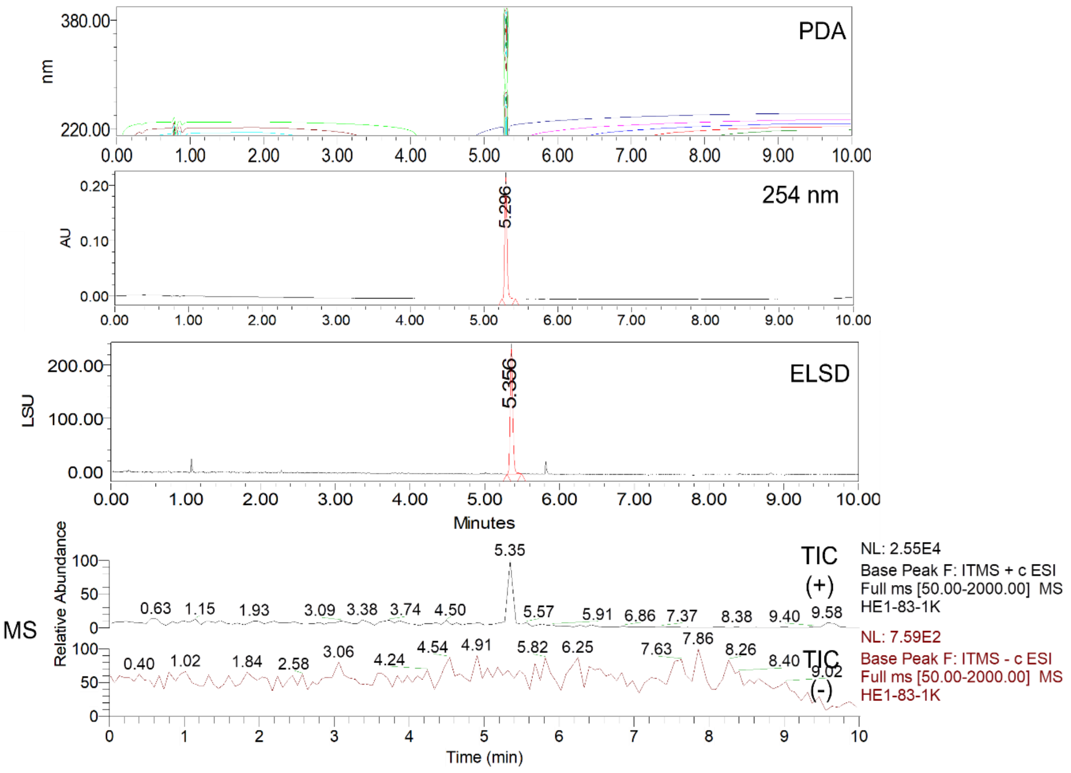

(B) sulochrin

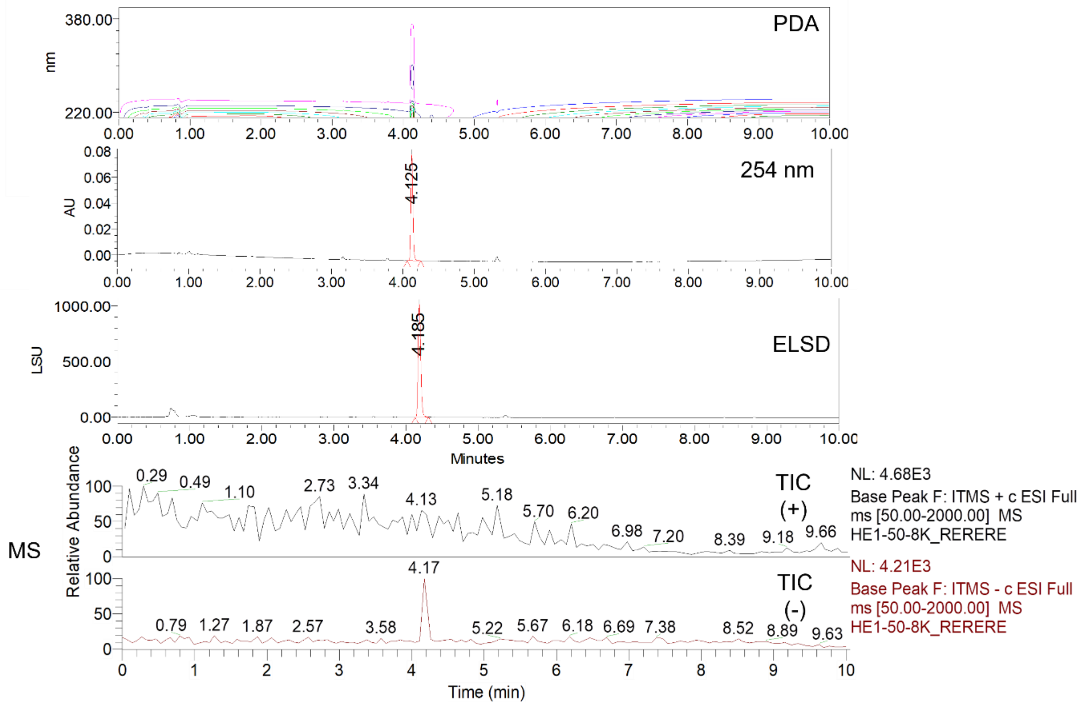

(C) citromycetin

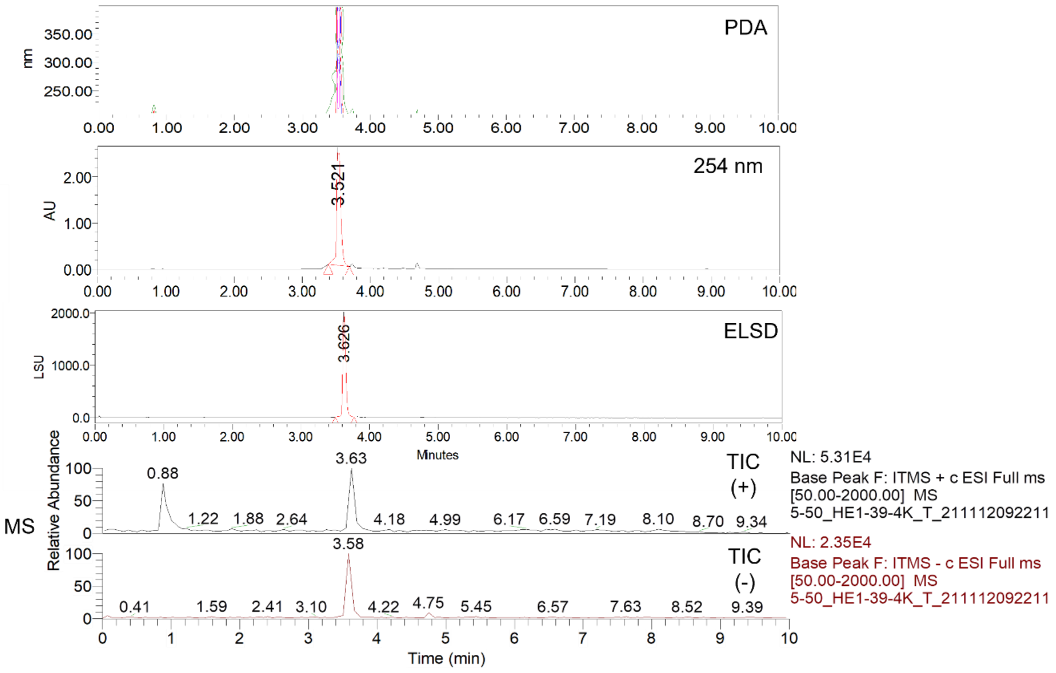

(D) citromycin

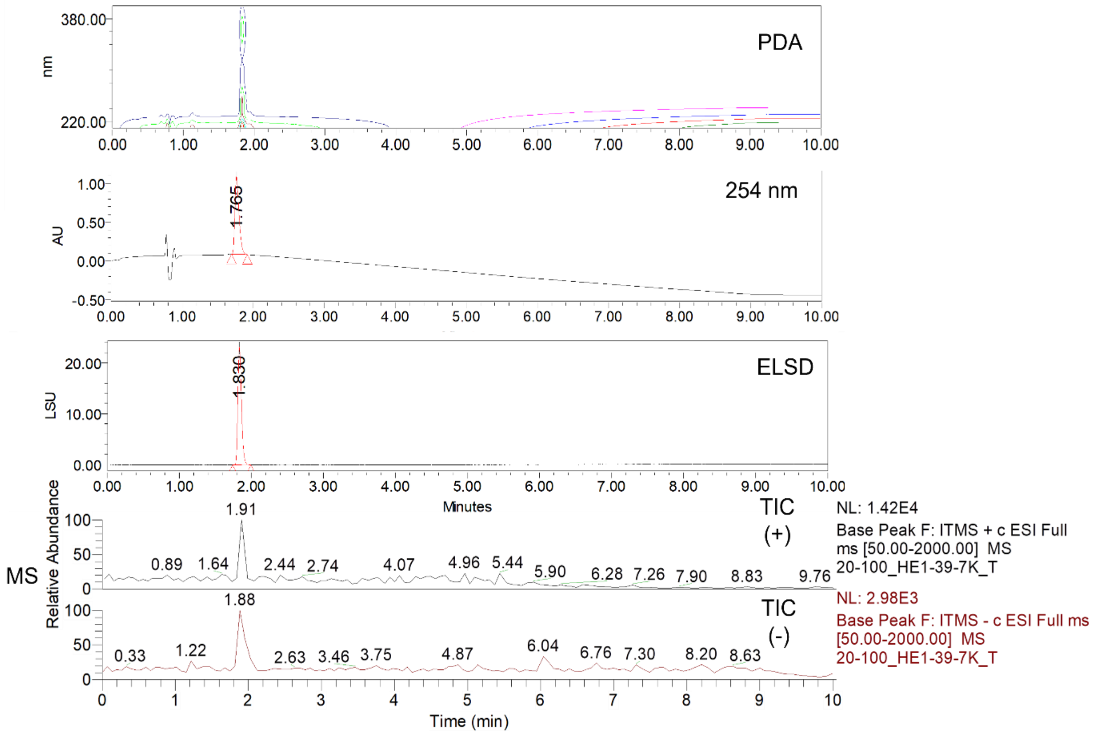

Figure S2. UPLC-PDA/UV (254 nm)/ELSD/MS chromatogram of penstyrylpyrone (A), sulochrin (B), citromycetin (C), and citromycin (D).

(A) penstyrylpyrone

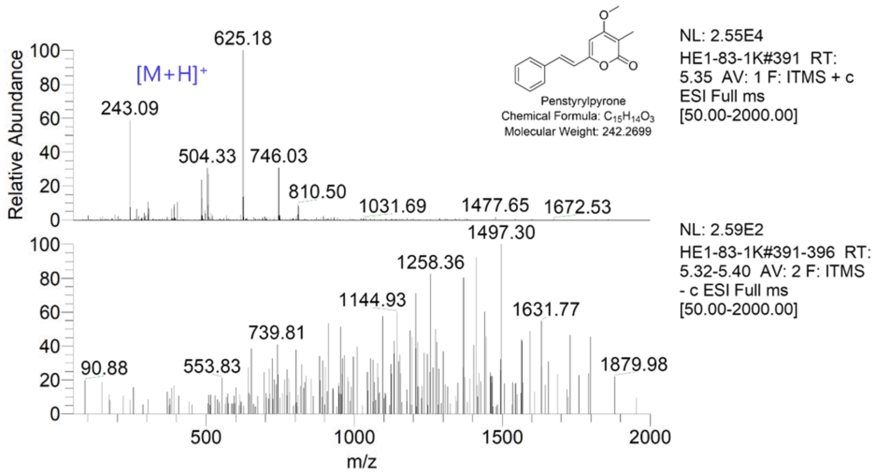

(B) sulochrin

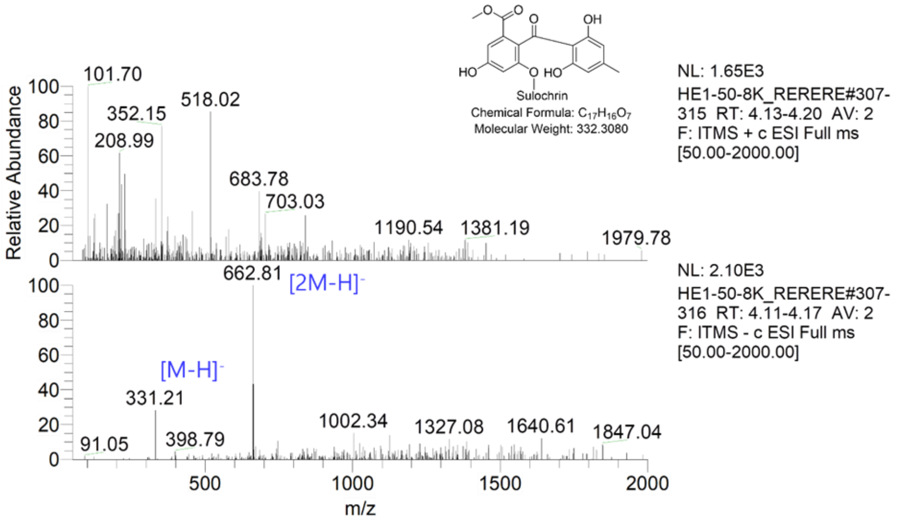

(C) citromycetin

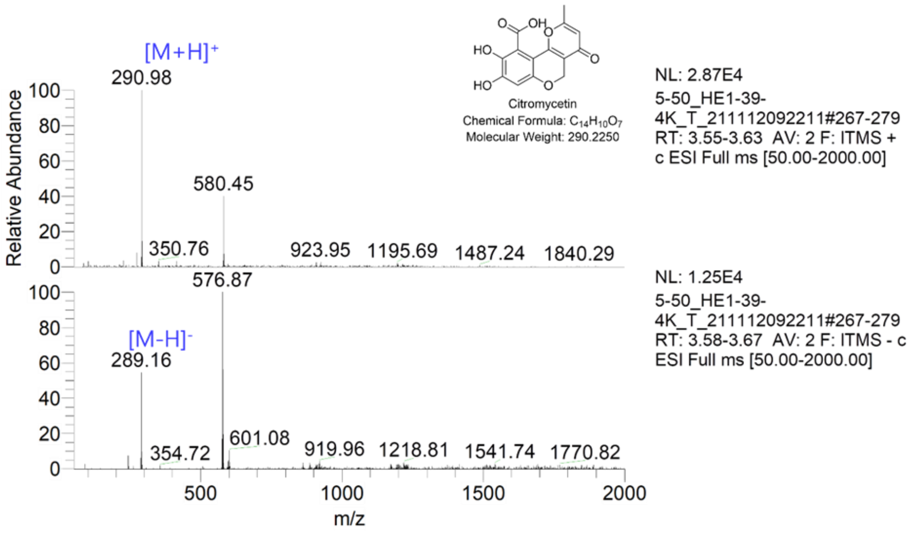

(D) citromycin

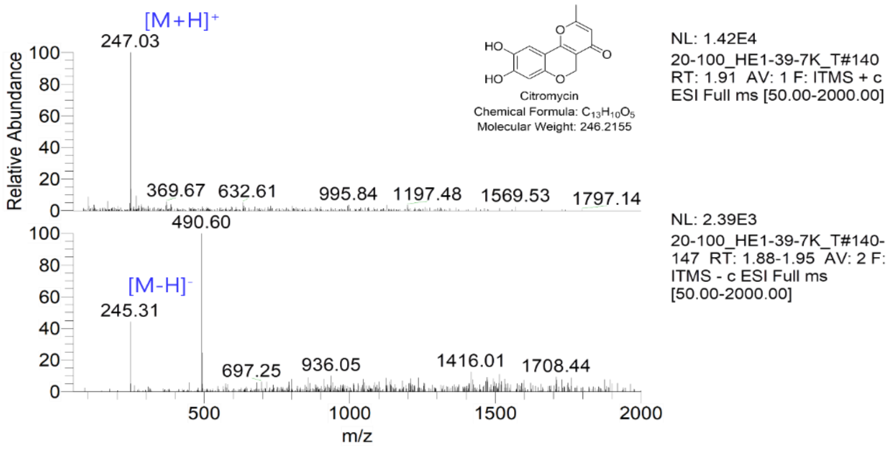

Figure S3. MS spectrum of penstyrylpyrone (A), sulochrin (B), citromycetin (C), and citromycin (D).

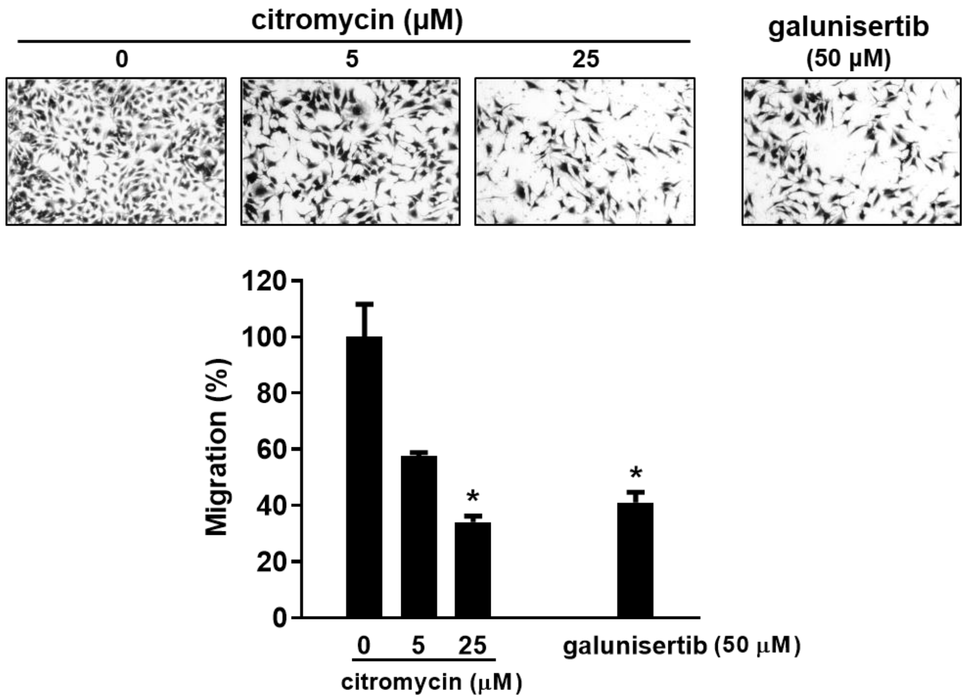

Figure S4. Comparison of the effects of citromycin and galnuisertib on the migration capacities of human ovarian cancer cells. A2780 cells were seeded in uncoated chambers for

migration assay and incubated for 24h in the presence or absence of citromycin and galunisertib, a potent inhibitor of TGFβ rector. Representative images of three independent experiments show the migratory cells. *P < 0.05, as determined by Student's t-test by comparison with untreated group.

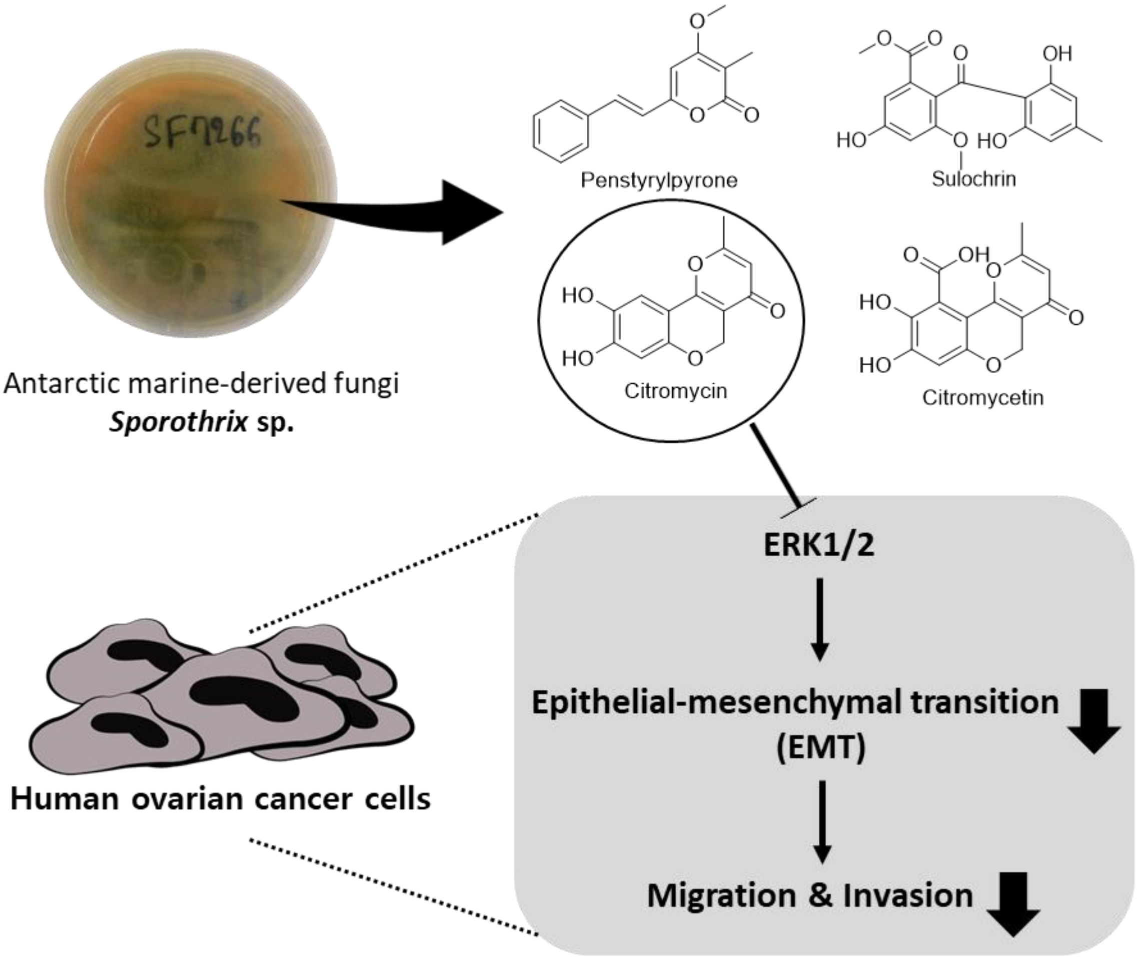

Figure S5. A schematic diagram summarizing the effect of citromycin on migration and invasion of human ovarian cancer cells
